# The effect of hyperbaric oxygen therapy on cognition, performance, proteomics, and telomere length—The difference between zero and one: A case report

**DOI:** 10.3389/fneur.2022.949536

**Published:** 2022-07-29

**Authors:** Joseph C. Maroon

**Affiliations:** Department of Neurological Surgery, University of Pittsburgh Medical Center, Pittsburgh, PA, United States

**Keywords:** hyperbaric, oxygen, physical, cognitive, enhancement, aging, anti-inflammatory

## Abstract

**Introduction:**

Hyperbaric oxygen (HBO2) therapy has recently been suggested for the treatment of different brain injuries as well as for physical and cognitive enhancement. The author recently carried out a self-experiment to obtain objective information on the effects of HBO2 therapy on neurocognition, cardiopulmonary function, neuroimaging and its effect on novel biomarkers such as telomere length and proteomics. In the following case report, the author will present and discuss the results and the differences between zero and one.

**Methods:**

This is a personal case report on a single subject, myself, who underwent a protocol of 60 daily HBO2 therapy sessions within 3 months. Pre- and post-therapy objective evaluation measured included computerized cognitive assessment, brain imaging, cardiopulmonary exercise test, physical assessments and blood tests including telomere length and proteomics.

**Results:**

Neurocognitive results showed a 3.1–3.8% improvements in global cognitive function as well as all other cognitive function domains. In the perfusion MRI, there was a relative increase ranging from 43.3 to 52.3% in cerebral perfusion in various areas subserving memory, coordination, and visual motor cortex function. Similar improvements in cerebral perfusion were seen in the SPECT scans, which ranged from 8.79 to 16.12% increased perfusion in the temporal pole and entorhinal cortex subserving memory, as well as in the subcallosal area and lingual gyrus. MRI-DTI showed prominent increases in fractional anisotropy in several white matter areas including 9% in the body of the corpus callosum, 16.85% in for the fornix and 22.06% in the tapetum. In the physical domains, there were improvements in both anaerobic threshold, exercise endurance, muscle strength, gait speed and grip strength in the 7–15% range. The telomeres length was doubled and clusters of inflammatory proteins dropped around the 40th session and remained low at the 60th session.

**Conclusion:**

The difference between zero and one in this single case study of HBO2 therapy confirmed improvement in objective biomarkers which measured cognition, memory, brain processing speed, athletic performance and neuroimaging modalities measuring cerebral perfusion, blood flow and tractography. Additional studies with larger sample size and randomized clinical trials using similar biomarkers are needed to confirm the results and to delineate the longevity of these improvements.

## Introduction

In 1976 William Sweet, professor and chairman of neurosurgery at Harvard, stated, “I believe we have become excessively imbued with the conviction that multiple consonant observations are necessary before even tentative conclusions can be drawn from biological phenomena, that a randomized statistically significant series is necessary for firm conclusions…we need to establish faith and the capacity of the biologist to reach a valid novel conception on the basis of one single set of facts. There is a staggering difference between zero and one when the one is a good idea” (1).

Hyperbaric oxygen (HBO2) therapy is one idea that has been FDA approved and is efficacious in the treatment of 13 different medical conditions including decompression sickness, non-healing wounds, severe anemia, carbon monoxide poisoning, among others (2). It also has been used off label for closed head injuries, post-stroke, post-traumatic stress syndrome (PTSD) and post-concussion syndrome (PCS) (3–7). Numerous HBO2 therapy mechanisms have been shown in both preclinical and clinical models including anti-inflammatory effects, stem cells recruitment, angiogenesis, neurogenesis, increased cerebral blood flow, mitochondrial function restoration, and cellular metabolism. By supplying repeated hyperoxic exposures, the hyperoxic-hypoxic paradox is utilized, increasing HIF-1a levels along with tissues hyperoxygenation (8–10). Recently, HBO2 therapy was shown to induce both cognitive and physical performance enhancements in healthy aging adults (11, 12). For 8 years, I have used HBO2 therapy to treat patients with PCS when all other treatment modalities had failed. Impressed with the results, I recently carried out a self-experiment to obtain objective information on the effects of HBO2 therapy on neurocognition, cardiopulmonary function, neuroimaging, telomere length, proteomics, among other objective measures. The author will present and discuss his results along the selected new biomarkers obtained pre- and post-therapy and the differences between zero and one.

## Materials and methods

### Hyperbaric oxygen therapy

A multiplace chamber accommodating up to 14 individuals was used for 60 individual therapy sessions over the course of 3 months with five sessions per week. HBO2 therapy was provided for 60 daily sessions using a class A multiplace chamber (Fink engineering, Australia). Protocol included total time of 2 h, with 100 min at pressure of two absolute atmospheres (ATA) (101 kPa). Additional 20 min were used for both compression and decompression at 1 meter/min. 100% oxygen was given at 20 min cycles, separated with 5 min of air, using a ResMed Quattro FX NV mask with continuous flow of 20–30 liters per minute. While at target pressure breathing oxygen, a validated cognitive training program was used for 20 min (i.e., in each of the 60 daily sessions) (13–15).

The following assessments were done at baseline and at the end of the therapy to compare objectively pre- and post-HBO2 therapy findings.

### Neurocognitive assessment

The following cognitive domains were assessed: memory, attention, information processing speed, coordination, planning and reasoning (executive function) and cognitive flexibility. Results were obtained from a computerized test, NeuroTrax battery (Mindstreams; NeuroTrax Corp, New York, NY) with alternative forms, and the MoCA (Montreal Cognitive Assessment) administered by a neuropsychologist 2 days prior to therapy and 3 weeks post-HBO2 therapy. NeuroTrax scores are standardized for age, gender and level of education.

### Physiological measurements

The following physiological parameters were evaluated by a certified exercise physiologist: cardiopulmonary exercise test (CPET, Cosmed, Italy) and spirometry (Cosmed, Italy). The CPET evaluates the integrative exercise responses across the pulmonary, cardiovascular, skeletal and muscular systems. Volumes of oxygen (VO2), volumes of carbon dioxide (VCO2), blood pressure, anerobic threshold (VO2AT) and heart rate are measured on a treadmill following the Boston Medical University protocols. Pulmonary function tests evaluated the standard forced volume capacity (FVC), forced expiratory volume In one second (FEV1) and their ratio (FV1/FVC).

Range of motion, time up and go test (TUG), 10-meter walk test, sit to stand test, Romberg balance, and tandem/semi-tandem balance tests were evaluated by a trained physical therapist. Additionally, resistance power in hand grip and knee extension were evaluated with dynamometers.

Finally, a timed competitive Olympic distance triathlon under similar topographic and atmospheric conditions was also undertaken before and after HBO2 therapy.

### Brain imaging

Brain imaging testing and details are summarized in Supplementary material 1.

### Blood sample for telomere and proteomics assessment

In addition to standard hematological studies, kidney and liver function tests, vitamin and hormonal assessments, leukocytes telomere length was determined pre- and post-therapy as delineated in the Supplementary material. Proteomic analysis was carried out at five different intervals before, during and after the therapy schedule (Supplementary materials 1, 2).

## Results

Baseline age was 81. Significant medical conditions included coronary artery disease with a history of a coronary artery bypass graft in 2013 and a coronary stent in 2018. Subjective complains were slight forgetfulness particularly with names and slowed brain processing speed. Exercise endurance was reduced as measured by triathlon endurance competition over 30 years. Sixty sessions of HBO2 therapy were well-tolerated with no adverse events. There were no significant changes in hematological, blood chemistry or body composition results. Subjective improvement included physical energy and ability to train longer and harder, along better thought process and processing speed.

### Neurocognitive and brain images changes

Neurocognitive results 3 weeks post HBO2 therapy (Table 1) showed a 3.1–3.8% improvements in global cognitive function as well as all cognitive function domains including memory, attention, information processing speed and executive function. Notably, there was a 27.1% improvement in delayed verbal memory.

**Table 1:** Cognitive changes 3 weeks post HBO2 therapy compared to 2 days prior to therapy.

**Domain**	**Relative change**
**Neurotrax**	
Global	3.10%
Memory	3.40%
Non-verbal memory	3.70%
Delayed verbal memory	27.10%
Attention	3.60%
Information processing speed	3.80%
Executive function	3.80%
MOCA	0

**Figure 1:**
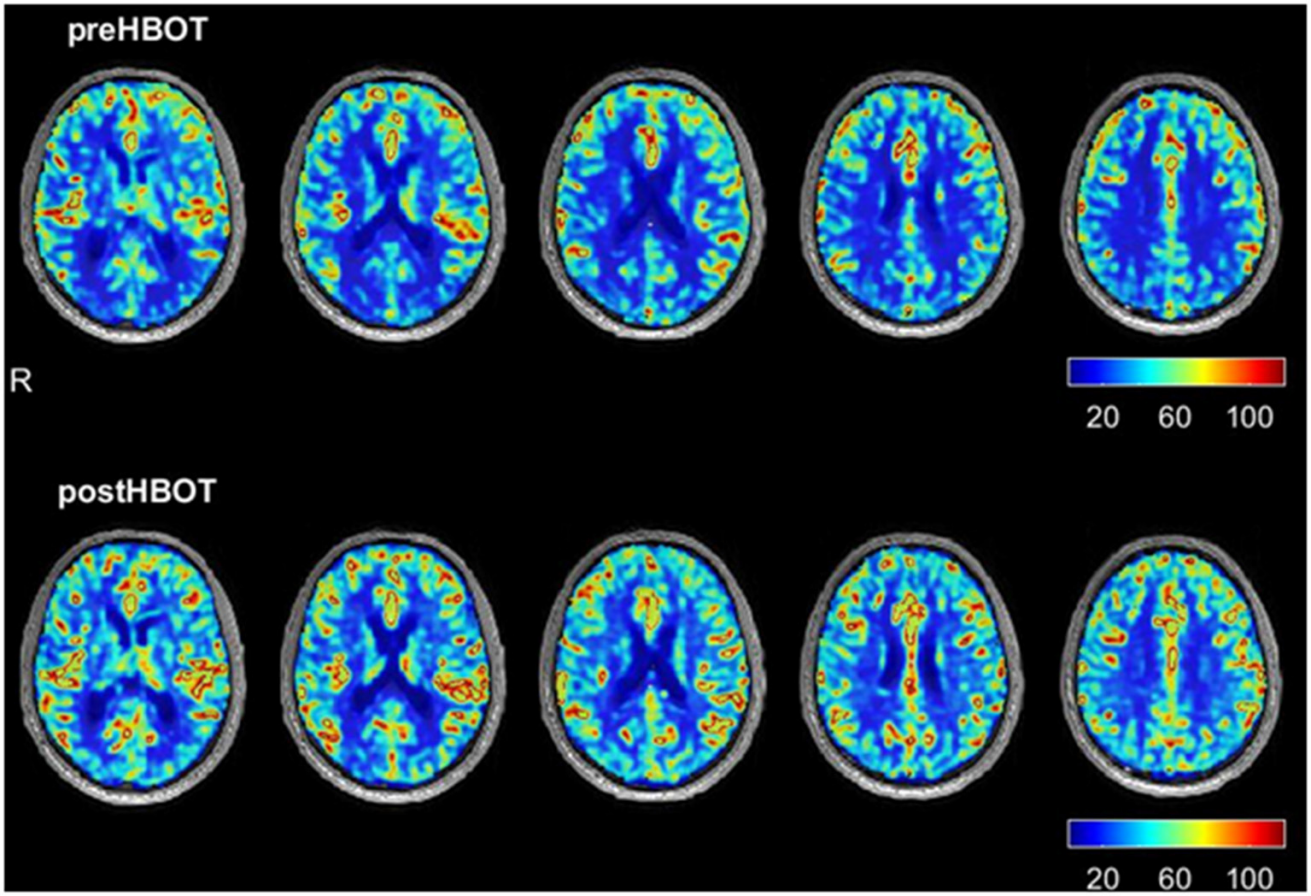
Brain MRI perfusion changes (DSC).

These results were associated with both MRI-DSC perfusion (Table 2; Figure 1), MRI-DTI (Tables 3, 4) and SPECT (Table 2) changes. There was a relative increase ranging from 43.3 to 52.3% in cerebral perfusion (Table 2) in various areas of subserving memory (entorhinal cortex, medial temporal gyrus), coordination (superior parietal lobule) and visual motor cortex function (lingual gyrus). There were no qualitative changes observed in FLAIR and SWI sequences.

**Table 2:** Brain perfusion changes (MRI-DSC).

**Brodmann area**	**Brain function**	**Baseline**	**Post**	**Relative change**
**SPECT**				
Brodmann 38 L	Emotion memory	0.76	0.88	16.12%
Brodmann 36 R	Memory	0.55	0.63	16.06%
Brodmann 36 L	Memory	0.7	0.81	15.22%
Brodmann 44 L	Language comprehension and speech	0.86	0.99	15.17%
Brodmann 25 L	Emotion regulation, decision making	0.82	0.92	13.06%
Brodmann 18 L	Secondary visual cortex	1.02	1.12	10.25%
Brodmann 17 L	Primary visual cortex	1.02	1.11	9.61%
Brodmann 11 L	Reward, emotion, connected to the Limbic system	0.8	0.87	8.79%
**MRI-perfusion (DSC)**				
Brodmann 28 R	Spatial memory, directionality	29.86	45.47	52.31
Brodmann 35 R	Visual memory	25.21	38.28	51.82
Brodmann 7 R	Visuo-motor coordination	25.13	37.42	48.89
Brodmann 17 R	Primary visual cortex	24.65	36.31	47.31
Brodmann 17 L	Primary visual cortex	26.17	38.4	46.74
Brodmann 27 R	Memory encoding and retrieval	32.15	46.19	43.66
Brodmann 2 L	Sensory perception, touch, texture	31.14	44.72	43.62
Brodmann 7 L	Visuo-motor coordination	26.87	38.5	43.33

**Table 3:** Brain white matter microstructure changes (MRI-DTIFA).

**Brain region**	**Brain function**	**29.06.2021**	**11.10.2021**	**% change**
**Most significant FA changes**				
Tapetum R	Connects the occipital lobes associated with processing visual information	0.16	0.19	22.06%
Fornix L	The fornix is important for learning and formation of new memories	0.21	0.24	16.85%
Superior fronto-occipital fasciculus R	Decision-making and cognitive processing of visual information	0.31	0.35	12.44%
Cerebral peduncle L	Refinement of motor function	0.45	0.49	10.68%
Cingulum (hippocampus) R	Learning, memory, behavior, sensation and perception	0.24	0.27	10.16%
Fornix R	The fornix is important for learning and formation of new memories	0.27	0.30	9.84%
Cingulum (cingulate gyrus) R	Learning, memory, behavior, sensation and perception	0.39	0.42	9.16%
Body of corpus callosum	Coordination of motor movements and touch sensation between the left and right	0.38	0.42	9.00%

**Table 4:** Brain gray matter microstructure changes (MRI DTI MD).

**Brain region**	**Brain function**	**29.06.2021**	**11.10.2021**	**% change**
**Most significant MD changes (X10** ^**−3**^ **)**				
Cerebral peduncle L	Refinement of motor function	1.63	1.44	11.33%
Genu of corpus callosum	Connect the prefrontal regions and is associated with decision-making, personality	1.30	1.16	10.74%
Superior fronto-occipital fasciculus R	Decision-making and cognitive processing of visual information	1.17	1.04	10.44%
Body of corpus callosum	Coordination of motor movements and touch sensation between the left and right	1.70	1.57	7.89%
Retrolenticular part of internal capsule R	Optic/visual pathway	1.16	1.07	7.77%
Posterior thalamic radiation R	Sensory and motor pathways, mainly visual	1.74	1.63	6.41%
Splenium of corpus callosum	Connects the occipital lobes associated with processing visual information	1.58	1.49	6.13%
Posterior corona radiata R	Cognition, decision-making, behavior regulation, motor movement and touch	1.24	1.17	6.10%

Similar improvements in cerebral perfusion were seen in the SPECT scans. These ranged from 8.79 to 16.12% increased perfusion in the temporal pole and entorhinal cortex subserving memory, as well as in the subcallosal area (BA25) and lingual gyrus (BA17, BA18). MRI-DT (Tables 3, 4) showed prominent increases in FA in several white matter areas including 9% in the body of the corpus callosum, 16.85% in for the fornix and 22.06% in the tapetum.

### Physiological changes

Physiological changes are summarized in Table 5. Improvement in anaerobic threshold and exercise endurance increased 10 and 15%, respectively. There was also a 7–10% improvement in muscle strength, gait speed and grip strength. No changes were noticed in body composition measures.

**Table 5:** Physiological changes.

**Domain**	**Relative change**
**Spirometry**	
Lung capacity	3.00%
**CPET**	
Exercise endurance	15.00%
Aerobic exercise capacity	2.93%
Anaerobic threshold	10.00%
Exercise power output	13.00%
**Physical tests**	
Fall risk (time up and go test)	29.00%
Muscle strength (sit to stand test)	7.00%
Gait speed (10 meters test)	10.00%
Grip strength (dynamometer)	10%

Following HBO2 therapy there was a 24 min or 9.2% improvement in the completion time of an Olympic distance triathlon (Table 6) that correlated with the improvement in the anabolic threshold and maximum volume of oxygen consumption. The changes in body composition are summarized in Table 7.

**Table 6:** Triathlon results.

	**July 2021**	**September 2021**	**Time**	**Relative**
	**race**	**race**		**change**
Total time	3:42:15	3:17:42	−24:33	−11.0%
TR1	6:32	7:35	+1:03	−15.3%
Bike	1:25:45	1:15:22	−10:23	−12.3%
TR2	2:49	1:46	−1.03	−32%
Run	1:30:39	1:25:51	−4:48	−5.5%

The triathlon consisted of 0.8 Km Swim, 32 Km Bike and 10 Km Run.

**Table 7:** Body composition changes.

	**Baseline**	**Post**	**Relative**
		**HBO2 therapy**	**change**
BMI	25.3	25.6	1.19%
Fat mass	5.2	5.2	0
Fat-free mass	20.1	20.3	1%
Total body water	38.11	38.51	1.04%
Visceral body tissue	1.51	1.51	0
Waist circumference	33	34	3.0%

### Telomeres

Following HBO2 therapy, there was an overall increase of 100% in the PBMC's telomeres length. Lymphocytes telomere length increased from 5.4 to 9 (66.7% change), while monocytes telomere length increased from 5.8 to 12.7 (119.9% change).

### Proteomics

**Figure 2:**
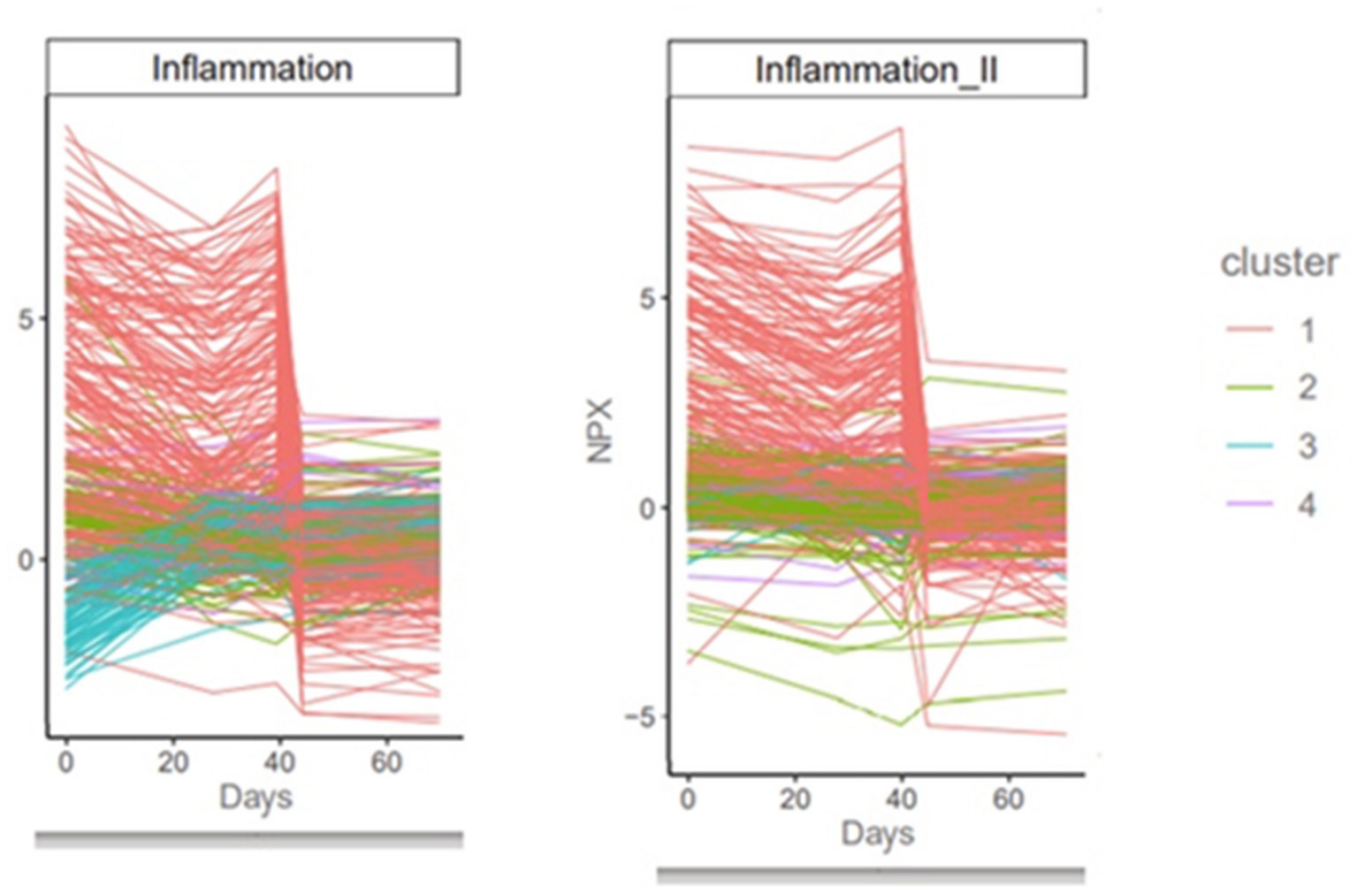
Proteomics results. The precipitous decrease in various clusters of inflammatory proteins around session 40 is consistent with the anti-inflammatory effect of HBO2 therapy as a function of the number of intermittent sessions and the hyperoxic-hypoxic paradox.

Clusters of inflammatory proteins dropped precipitously around the 40th session and remained low at the 60th session (Figure 2; Supplementary material 2). No other clusters showed a similar trend.

## Discussion

HBO2 therapy is a well-established treatment modality for non-healing wounds, radiation injuries as well as different hypoxic or ischemic events (such as carbon monoxide toxicity, infections, among others). In the past decade, evidence has accumulated on the benefits of HBO2 therapy in different types of brain injuries including stroke and traumatic brain injury (TBI) (3–7). Recently, by using a novel protocol, it has been evaluated for enhancement of both cognitive and physical capabilities in healthy adults (11). The use of HBO2 therapy for the enhancement of athletic performance rather than the treatment of injury/pathology has also been recently suggested. In this single case report, the results provide additional support of HBO2 therapy effect on neurocognition, physical performance and biomarkers.

The neurocognitive changes in this case are consistent with the Hadanny et al. previous study on cognitive function enhancement in healthy adults with HBO2 therapy. In their study, the global cognitive score improved ~5% with all domains affected including memory, executive function, attention and information processing speed (16). Interestingly, delayed verbal memory did not show a significant interaction in their findings compared to my notable change. Similarly, in the mentioned randomized clinical trial, there were significant changes in brain perfusion, where Broadman area 7 is shared. However, in this case, there were several additional temporal and mesial regions with increased perfusion, which may be related to the notable change in memory. These changes in imaging findings including SPECT, MRI-DSC, and DTI were correlated with objective improvements in the author's cognitive and physiological domains. DTI characterizes the diffusion of water in the tissues; thus, it indicates microstructural density, spacing, and orientational organization of cell membranes, including myelin. In older healthy subjects, both FA and MD show overall good test-retest reliability and reproducibility (17). The author's changes in both FA and MD following HBO2 therapy reflect a significant change in brain tissue microstructure integrity and suggest actual neuroplasticity.

The physiological improvements in this case are consistent with a recent study in which HBO2 therapy effects on endurance and athletic performance were evaluated (12). In this recent double blind randomized placebo-controlled study on 37 healthy middle aged master athletes, improvements were observed in maximal oxygen consumption, power and anaerobic threshold. Muscle biopsies confirmed mitochondrial biogenesis related to the HBO2 therapy and provided an explanation for the performance enhancement. The telomere changes in this case were even more notable than previously reported. Hadanny et al. reported an average of 25–37% increase in lymphocyte telomere length in a group of 35 healthy elderly (18). Telomere elongation is believed to be due to the novel HBO2 therapy protocol which activates the hyperoxic/hypoxic paradox (19). Through intermittent relative hypoxic exposure, the transcription factor, hypoxia inducible factor (HIF-1a), is expressed which triggers many cellular processes including increased neovascularization *via* VEGF and telomere elongation (18). We hypothesize that HIF-1a is triggered over time using the protocol activating the hyperoxic/hypoxic paradox, however, no studies so far, including this one, have measured and demonstrated this association in humans *in vivo*.

The encouraging initial data on proteomics is consistent with hypotheses around the biological anti-inflammatory impact of HBO2 therapy after a certain number of repeated intermittent sessions. While this is only a single case report and more data is needed to draw conclusions, there is certainly a cluster of proteins that do move in response to HBO2 therapy, with a potential indication of lowering inflammatory proteins/response as a function of time and the number of sessions. This interesting finding warrants further investigation.

This current report has obvious limitations related to being a single case and potential personal positive bias based on the author's experience with managing PCS patients. However, objective neuropsychological testing, which used alternative forms were utilized in order to minimize learning effect. The test-retest validity of these tools has been thoroughly validated previously in the literature (20–22). Although considered as the gold standard of evidence-based medicine, randomized controlled trials have several disadvantages. First, they are conducted on very selective populations managed in tightly controlled settings, which may not truly represent real world data. Second, in most cases, these studies report group effects, where the individual effect can vary significantly. Post market studies and reports serve as a good example of such instances where a drug may have been proven beneficial in group statistics but harmful in a few cases. Therefore, the usefulness of an individual case report like this, should not be underestimated because of small sample size when biomarkers provide objective support of the results. However, future studies with large sample size are needed to delineate such effects in a large scale.

Full exertion to exhaustion was carried out before and after HBO2 therapy, however, this is subjective and could be affected by several factors including diet and rest although no changes were made in these parameters. Another limitation is the unknown long-term effects. It is yet to be determined whether additional therapy sessions will be required to maintain these improvements and avoid subsequent deterioration. Further studies are required in this direction. Lastly, this therapeutic program requires substantial commitment in terms of time and financial resources not available to all.

## Conclusion

The difference between zero and one in this single case study of HBO2 therapy confirmed improvement in objective biomarkers, which measured cognition, memory, brain processing speed, athletic performance and neuroimaging modalities measuring cerebral perfusion, blood flow and tractography. While such observations were noted in a single case report, they show promising results, and additional studies with larger sample size and randomized clinical trials using similar biomarkers are needed to confirm these results and delineate the longevity of these improvements.

## Data availability statement

The raw data supporting the conclusions of this article will be made available by the authors, without undue reservation.

## Ethics statement

Ethical review and approval was not required for the study on human participants in accordance with the local legislation and institutional requirements. The patients/participants provided their written informed consent to participate in this study.

## Author contributions

JM conceived, designed the report as well as wrote the manuscript.

## Funding

This work was supported in part by grants to the Neuroscience Research Foundation from the following philanthropists: Alba Tull, Lewis Topper, Ray Pronto, Dennis Heindl, Nelson Peltz, John and Kathy Garcia, Carl Campbell, and Coury Financial Inc. The funders were not involved in the study design, collection, analysis, interpretation of data, the writing of this article or the decision to submit it for publication.

## Conflict of interest

Author JM is affiliated with the following: The NFL Head, Neck and Spine Committee, Consultant to the Pittsburgh Steelers, and Medical Director of World Wrestling Entertainment (WWE).

## Publisher's note

All claims expressed in this article are solely those of the authors and do not necessarily represent those of their affiliated organizations, or those of the publisher, the editors and the reviewers. Any product that may be evaluated in this article, or claim that may be made by its manufacturer, is not guaranteed or endorsed by the publisher.
