# Supplementary material for: The effect of hyperbaric oxygen therapy on cognition, performance, proteomics, and telomere length—The difference between zero and one: A case report
Source: Front Neurol. 2022 Jul 29;13:949536. doi: 10.3389/fneur.2022.949536 (PMC9373903; doi:10.3389/fneur.2022.949536)
Supplement: Supplementary file 2 [file Table_2.DOCX]

Brain Magnetic Resonance Imaging (MRI)

Images were acquired on MAGNETOM Vida 3T Scanner, configured with a 64-channel receiver head coils (Siemens Healthcare, Erlangen, Germany). The MRI protocol included the following sequences: high resolution MP-RAGE 3D T1-weighted, diffusion tensor imaging (DTI) sequence in 64 gradient direction, b-value of 1000 sec/mm2, dynamic susceptibility contrast enhancement (DSC) and post-contrast high-resolution MP-RAGE 3D T1-weighted images. The MRI protocol also included T2-Fluid-attenuated inversion recovery (FLAIR), and susceptibility weighted imaging (SWI), using standard parameters, for clinical brain evaluation.

MRI-Dynamic Susceptibility Contrast (DSC)

Whole-brain quantitative perfusion imaging was be performed using DSC-MRI technique. DSC-MRI scans were acquired using 50 T2* weighted gradient-echo planar imaging (EPI) volumes. Following 2 baseline volumes, a single dose injection of Gadoteric acid solution is applied (Cyclolux 0.5 mmol/ml, 0.2 ml/kg, administered at 5 ml/sec). Brain perfusion was analyzed based on whole brain quantitative cerebral blood flow (CBF), cerebral blood volume (CBV), and mean transient time (MTT) maps as described in Ostergaard’s study (1) using in-house software written in Matlab R2018b (Mathworks Natick, MA). Scan parameters: TR: 2,500 ms, TE: 30 ms, flip angle: 30°, voxel size: 1.8 x 1.8 mm, matrix: 128 x 128, number of slices: 36, and slice thickness: 3.9 mm, with a total scan time of 2.2 min.

MRI-DTI

Microstructure changes in gray and white matter were determined using diffusion tensor imaging (DTI) sequence in 64 gradient direction and b-value of 1000 sec/mm^2^ and 5 repetitions of b=0 volumes. The fractional anisotropy (FA), mean diffusivity (MD), and the parallel and radial diffusivities maps were analyzed using voxel-based analysis. Scan parameters: TR: 3,400 ms, TE: 72 ms, voxel size: 2 mm^3^, FOV of 248 mm, number of slices: 60, simultaneous multi slice (SMS) factor: 3, with a total scan time of 4.2 min.

Preprocessing of DTI images was preformed using the SPM software (version 12, UCL, London, UK) and included motion correction, co-registration with MPRAGE T1 images, spatial normalization, and spatial smoothing with kernel size of 6 mm full width half maximum (FWHM). Diffusion brain volume denoising was performed using the joint anisotropic LMMSE filter for stationary rician noise removal and calculation of DTI-FA (fractional anisotropy) and MD (mean diffusivity) maps were performed using in house software written in MATLAB R2021b (MathWorks, Natick, MA) (2).

Brain Single Photon Emission Computed Tomography (SPECT)

Brain activity was assessed using single photon emission computed tomography (SPECT) 2 days prior to and 10 days after the program. The imaging was conducted using 925–1,110 MBq (25–30 mCi) of technetium-99m-methyl-cysteinate-dimmer (Tc-99m-ECD) at 40–60 min post injection using a dual detector gamma camera (Siemens Medical Systems) equipped with high resolution collimators. Data were acquired in 3-degree steps and reconstructed iteratively with Chang method (μ= 0.12/cm) attenuation correction (3).

Both SPECT studies were normalized to the median brain activity in the entire brain and were then reoriented into Talairach space using NeuroGam (Segami Corporation) to identify Brodmann cortical areas and in order to compute the mean perfusion in each Brodmann area (BA). In addition, volume rendered brain perfusion images were reconstructed and normalized to maximal cerebellum activity. Changes in perfusion in all Brodmann areas was determined by calculating the percentage of the difference of the normalized activity value between post-treatment and pre-treatment divided by the pre-treatment value.

Telomeres

Whole blood samples were collected into BD CPT vacutainer tubes with Sodium Citrate using a standard technique, at baseline and on the last day of HBO2 therapy. Whole blood was diluted using phosphate buffered saline (PBS). Following centrifugation, the cell layers (buffy coat) were immediately collected via pipette and transferred to 50 mL conical centrifuge tubes, resuspended with sufficient 1X PBS to a volume of 50 mL and centrifuged at 300×*g* for 10 min at 25° C degrees.

Telomeres were labelled according to the Dako PNA/FITC kit protocol (Code K5327). On a single cell suspension consisting of a mixture of peripheral blood mononuclear cells (PBMCs) and TCL 1301 cell line (control cells), the DNA was denatured for 10 minutes at 82° C in a microcentrifuge tube either in the presence of hybridization solution without probe or in hybridization solution containing the fluorescein-conjugated PNA telomere probe. The hybridization took place in the dark at room temperature (RT) overnight. The hybridization was followed by two 10-minute post-hybridization washes with a wash solution at 40° C. The sample was then labeled with CD4+, CD8+, CD3+, CD19+ and CD56+ conjugated antibodies in an appropriate buffer for further flow cytometric analysis. Each sample was run in duplicate. Following flow cytometric analysis, the relative telomere length (RTL) was calculated for the entire PBMC population as well as the subpopulations: CD3+/CD4+ (T-helper), CD3+/CD8+ (T-cytotoxic), CD3+/CD56+ (natural killer) and CD19+ (B-cells). The RTL value was calculated as the ratio between the telomere signal of each sample and the control cell (TCL 1301 cell line) with correction for the DNA index of G0/1 cells. Sample cells and control cells were analyzed separately for DNA ploidy using propidium iodide staining to standardize the number of telomeres ends per cell and thereby telomere length per chromosome.

Proteomics

Blood samples were drawn at five different intervals coinciding with the number of sessions: baseline, session 15, session 30, session 40 and the final session (60). The plasma was immediately separated and stored at -80° C until analyzed. The proteins expressed represent the inflammatory proteins IL-1beta, IL-6 and TNF-alpha. The Olink PEA assay uses matched pairs of antibodies to bind their target protein with high specificity. The matched pairs of antibodies are labeled with complementary DNA oligo sequences. When the pair of antibodies bind the intended target protein the complementary DNA sequences combine, hybridize, and create a DNA amplicon that is that is read out on a Sequencer to provide the protein measurement (4).

Body Mass Composition

In addition to a full nutritional assessment, the following were determined: body mass index (BMI), fat mass, fat free mass, skeletal muscle mass, body composition, total body water, bioelectrical impedance of fat free mass and visceral adipose tissue and the phase angle—a measure of the quantity and quality of somatic cells which is an assessment of nutritional condition and state of health.

**References:**

1. Ostergaard L, Weisskoff RM, Chesler DA, Gyldensted C, Rosen BR. High resolution measurement of cerebral blood flow using intravascular tracer bolus passages. Part I: Mathematical approach and statistical analysis. *Magn Reson Med* (1996) 36:715–725. doi: 10.1002/mrm.1910360510

2. Tristán-Vega A, Aja-Fernández S. DWI filtering using joint information for DTI and HARDI. *Med Image Anal* (2010) 14:205–218. doi: 10.1016/j.media.2009.11.001

3. Jaszczak RJ, Chang LT, Stein NA, Moore FE. Whole-body single-photon emission computed tomography using dual, large-field-of-view scintillation cameras. *Phys Med Biol* (1979) 24:1123–1143. doi: 10.1088/0031-9155/24/6/003

4. Wik L, Nordberg N, Broberg J, Björkesten J, Assarsson E, Henriksson S, Grundberg I, Pettersson E, Westerberg C, Liljeroth E, et al. Proximity Extension Assay in Combination with Next-Generation Sequencing for High-throughput Proteome-wide Analysis. *Mol Cell Proteomics* (2021) 20:100168. doi: 10.1016/j.mcpro.2021.100168
